# Adnexal Torsion in the Third Trimester

**DOI:** 10.7759/cureus.60836

**Published:** 2024-05-22

**Authors:** Subanhey Thiyagalingam, Chiara Petrosellini, Claire Mellon

**Affiliations:** 1 Obstetrics and Gynaecology, North Middlesex University Hospital, London, GBR; 2 Obstetrics and Gynaecology, University College Hospital, London, GBR; 3 Obstetrics and Gynaecology, Whittington Health NHS Trust, London, GBR

**Keywords:** emergency surgery, pregnancy, high-risk pregnancy, third trimester, ovarian pathology, adnexal torsion

## Abstract

A 41-year-old woman presented with acute, severe left-sided abdominal pain and vomiting at 37 weeks’ gestation. Her symptoms were attributed to renal colic, and she was admitted for supportive treatment. During her admission, she went into spontaneous labour. Due to other obstetric indications, the team proceeded with delivery by emergency caesarean section. Intra-operatively, a necrotic left fallopian tube and ovary were identified, and a diagnosis of adnexal torsion (AT) was recognised. There was no return of tissue perfusion on de-torsion, and a left salpingo-oopherectomy was performed. AT in pregnancy is unusual, with only a minority of cases occurring in the third trimester. This is a challenging diagnosis to establish and requires a high index of suspicion. Ultrasound and magnetic resonance imaging can be helpful in establishing a diagnosis but should not delay definitive treatment. Prompt surgical intervention is paramount to prevent irreversible damage to ovarian tissue.

## Introduction

Adnexal torsion (AT) is an emergency in which the ovary, and sometimes the fallopian tube, twists upon its ligamental supports. This compromises the adnexal blood supply, resulting in tissue ischaemia and ovarian necrosis. Prompt diagnosis and management are imperative as delay can result in permanent damage to the reproductive organs with local and systemic sequelae.

AT is uncommon, with a reported incidence of six in 100,000 per year [1]. Diagnosis is particularly challenging in pregnancy, owing to the distortion of abdominal anatomy by the gravid uterus, the difficulty of abdominal examination during pregnancy, and the wide range of potential diagnoses associated with its non-specific symptoms [2].

It is estimated that 12-18% of all cases of AT occur during pregnancy [3], with only 19% of these occurring in the third trimester [2]. Whether pregnancy itself represents a risk factor for AT is contentious, with some evidence that pregnancy may even be protective [2,4,5]. When AT does occur in pregnancy, being in the first trimester or early second trimester [2,6,7], ovarian hyperstimulation secondary to assisted reproductive technology [2,8], and the presence of pre-existing ovarian masses greater than 5 cm in diameter [4,7] are thought to be the most significant risk factors.

This case was previously presented as a poster at the Royal College of Obstetricians and Gynaecologists (RCOG) World Congress on June 12, 2023.

## Case presentation

A 41-year-old parous woman presented to emergency maternity services with abdominal pain at 37 weeks’ gestation. She previously had two second-trimester miscarriages with failure of a McDonald cervical cerclage, leading to the insertion of a transabdominal cervical cerclage. Subsequently, she had a caesarean delivery of twins at term, in which she was noted to have bilaterally enlarged ovaries with a polycystic appearance. A postnatal ultrasound and serial Ca-125 serum levels had been reassuring, and no further follow-up was arranged.

**Figure 1:**
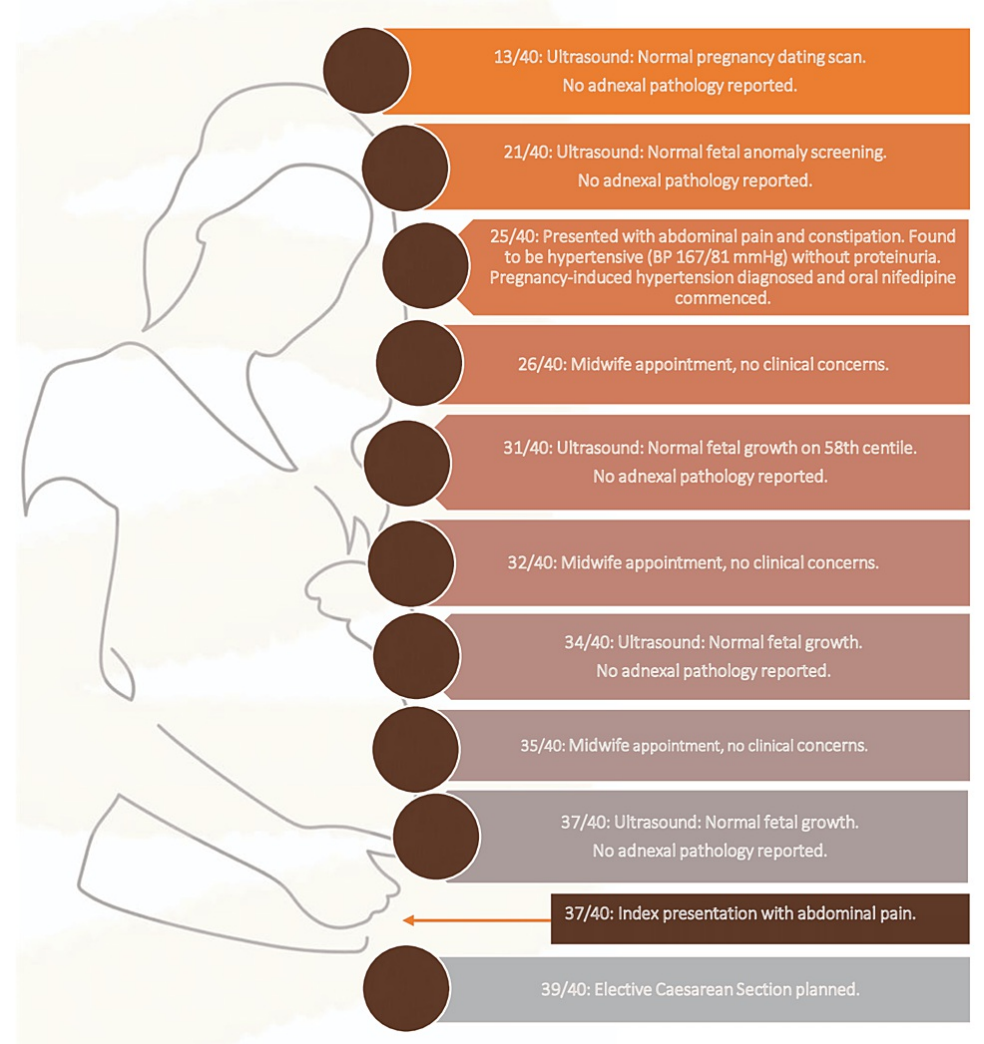
Timeline of antenatal care received n/40 represents the gestational age as 'n'th week out of 40 weeks. Image credits: Author (Subanhey Thiyagalingam)

The index pregnancy was a spontaneous conception. She developed pregnancy-induced hypertension (PIH) at 25 weeks and was commenced on oral antihypertensive medication. Regular urine dipstick tests were negative for protein and serial urinalyses for urine protein-creatine ratio (U-PCR) were below the diagnostic threshold for pre-eclampsia. Blood pressure readings both at home and in the clinic remained stable within the target range. Her antenatal course is summarized in Figure 1. A repeat elective caesarean section had been arranged for 39 weeks’ gestation.

She presented to maternity services 12 days prior to her caesarean section date, describing a four-hour history of severe, constant, left-sided, abdominal back pain. This was of a sharp and cramping nature, radiating from loin to groin and associated with repeated vomiting. She denied any vaginal bleeding or watery loss, lower urinary tract symptoms, change in bowel habits, or fever. She reported good fetal movements. On arrival, she was hypertensive, but the other vital signs were normal (Table 1).

**Table 1:** Initial maternal observations on arrival

Observation	Value
Blood pressure (mmHg)	152/81
Heart rate (beats per minute)	74
Respiratory rate (breaths per minute)	16
Oxygen saturation (%)	100% without supplemental oxygen
Temperature (°C)	36.4

On examination, her abdomen was soft but there was generalised tenderness to palpation and some left renal angle tenderness. There was no palpable uterine activity and her cardiotocogram (CTG) was classified as normal. A speculum examination was unremarkable, indicating that she was not in labour. Blood test results on admission are shown in Table 2. An initial urine dipstick test showed leucocytes and ketones and was sent for microscopy, sensitivity and culture. This later showed mixed growth of three organisms, whilst microscopy showed scanty white cells, no red cells and numerous epithelial cells. There was no proteinuria. Her blood pressure normalised quickly following oral nifedipine. Given the site and nature of her pain, renal colic was suspected. She was admitted for further monitoring, analgesia, and antiemetics, to organise an abdominal ultrasound the following day. It is important to note that the patient was admitted out of hours, and immediate access to ultrasound imaging was not available.

**Table 2:** Blood test results on admission

Blood test	Result	Reference Range
Haemoglobin (g/L)	116	11–165
White cell count (x 10^9^/L)	10.3	3.5–12
Neutrophils (x 10^9^/L)	9.1	1.7–7.5
Platelets (x 10^9^/L)	194	140–400
C-reactive protein (mg/L)	2	0–5
Amylase (IU/L)	113	28–100
Urea (mmol/L)	3.4	2.1–7.1
Creatinine (µmol/L)	54	49–92
Sodium (mmol/L)	138	135–145
Potassium (mmol/L)	4.5	3.5–5.1

On clinical review a few hours later, the pain had partially improved with analgesia and antispasmodics. There had been no further vomiting. During repeat abdominal examinations, there continued to be no evidence of peritonism and no palpable uterine activity. Blood pressure readings had remained within normal range and all other observations remained stable.

Possible causes of abdominal pain in the third trimester are summarised in Table 3. Obstetric differentials, in this case, included labour, placental abruption, pre-eclampsia, and uterine rupture. On initial clinical examination, uterine contractions were not palpable, and the cervix was closed on speculum examination, albeit with an abdominal cerclage in situ. Placental abruption and uterine rupture were excluded on the basis of a soft abdomen on palpation, normal maternal observations, and a normal CTG. Pre-eclampsia was excluded on the basis of no proteinuria and reassuring initial blood results.

**Table 3:** Differential diagnoses for abdominal pain in the third trimester

Obstetric	Non-obstetric
Labour at term or preterm	Adnexal mass, torsion or cyst accident
Uterine rupture	Urinary tract infection
Placental abruption	Renal and/or ureteric calculi
Pre-eclampsia (haemolysis, elevated liver enzymes and low platelet count - HELLP syndrome)	Appendicitis
Acute fatty liver of pregnancy	Gastroenteritis
Chorioamnionitis	Pancreatitis
Pelvic girdle pain	Cholelithiasis and/or cholecystitis
	Diabetic ketoacidosis
	Constipation
	Traumatic injury

The absence of urinary symptoms or systemic features suggested that a urinary tract infection, considering the level of pain, was unlikely. The absence of blood in the urine dipstick test made renal tract calculi unlikely. However, it is important to note that 10% of calculi can present without microscopic haematuria [9], and imaging is therefore required to exclude this diagnosis.

The absence of fever or change in bowel habits in the presence of normal inflammatory markers made appendicitis, cholecystitis, pancreatitis, gastroenteritis, and pelvic inflammatory disease less likely. Despite the acute onset of pain, bowel obstruction or perforation did not seem likely given the history of normal bowel habits and the absence of peritonism. It must be noted, however, that identifying peritonism on examination of a heavily gravid abdomen can be challenging. Ovarian cyst accidents and AT are rare in the third trimester [2,10], particularly when ovarian enlargement is not known. Nevertheless, these are important differentials to consider, particularly if other possible diagnoses have been excluded.

The following morning, the patient developed regular uterine contractions. The CTG performed was classed as suspicious, raising concerns for fetal wellbeing. A plan for urgent delivery via caesarean section was made, necessitated by her trans-abdominal cerclage, and imaging to investigate the original presenting complaint had to be delayed.

An uncomplicated caesarean section was performed under spinal anaesthesia. Intra-operatively, copious free fluid was noted in the abdomen. A male infant weighing 2,992 g was delivered in excellent condition.

**Figure 2:**
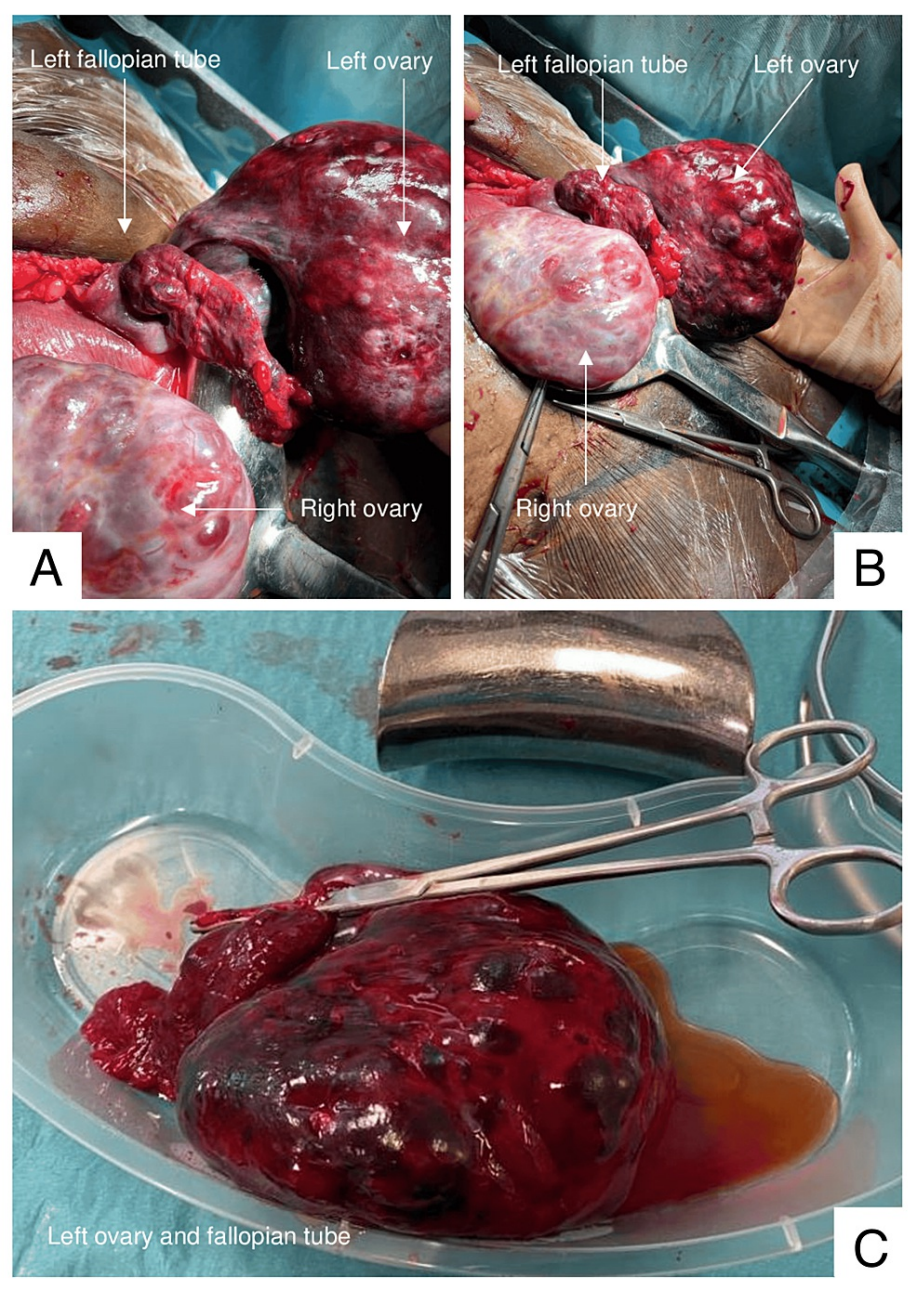
Intra-operative images Image panel A: Image of left adnexal torsion and the necrotic appearance of the left fallopian tube and ovary identified at laparotomy. Image panel B: Image of bilateral polycystic and enlarged ovaries with left adnexal torsion. Image panel C: Image of the left fallopian tube and ovary in the kidney dish following salpingo-oopherectomy.

On further inspection of the pelvis, both ovaries appeared polycystic and enlarged, each measuring approximately 10 x 10 cm. The left fallopian tube and ovary were twisted four times around their pedicle and appeared necrotic (Figure 2). The right ovary, whilst also enlarged, appeared healthy and well-vascularised. Upon untwisting of the left adnexa, there was no return of blood supply. The patient was informed of the findings, and verbal consent was obtained to proceed with the left salpingo-oopherectomy. Aspiration was used to decompress the follicles on the right ovary in order to reduce the overall ovarian size and minimise the immediate risk of contralateral AT.

The patient had an uncomplicated postnatal recovery. Outpatient blood pressure monitoring was arranged via her community midwife and general practitioner. Histopathological assessment of the intraoperative specimen showed widespread congestion, haemorrhage, and oedema, in keeping with ovarian torsion. The presence of luteinized cells was noted, consistent with pregnancy. There was no evidence of malignancy or atypia.

## Discussion

This case illustrates that recognition of AT in pregnancy can be challenging, even in the presence of classical signs and symptoms. AT typically presents with acute-onset abdominal pain, which may be associated with nausea and/or vomiting [2-4]. A raised white cell count (WCC) may also be seen in AT [11]. However, this may have limited diagnostic utility in pregnancy as physiological elevation in WCC is common [12].

Whilst the clinical features of AT in pregnant and nonpregnant women are similar, pregnant women tend to present earlier following the onset of pain and are twice as likely to have recurrent AT [8]. A high index of suspicion is therefore required in pregnant women who repeatedly present with abdominal pain.

In this case, it is possible that the obstetric team were falsely reassured by the absence of an adnexal mass on previous pregnancy ultrasounds. Over the course of this pregnancy, a total of six ultrasounds were performed, none of which commented on adnexal structures. It is important to note that ovarian morphology may not universally be reported in dating and anomaly screening ultrasounds as this is not a requirement in the United Kingdom. A previous history of ovarian enlargement, however, should have been recognised antenatally, triggering further suspicion for adnexal pathology.

The typical features of AT on ultrasound include unilateral ovarian enlargement, ovarian oedema, abnormal positioning of the adnexa, reduced or absent blood flow to the ovary, the ‘whirlpool’ sign of twisted vessels, and pelvic free fluid [6]. Despite these recognised radiological features, ultrasound may be less reliable in the diagnosis of AT in pregnancy compared to the general population [13]. In cases where ultrasound findings are inconclusive, magnetic resonance imaging (MRI) should be considered as there is growing evidence that it can support the diagnosis of AT [10]. MRI can clarify the origin and nature of a mass to assist clinical decision-making. This is particularly important where factors such as bowel gas shadowing, obesity, and anatomical distortion from pregnancy may limit ultrasound studies.

Delayed recognition of AT in this case is likely due to non-specific symptoms, in combination with reassuring blood tests and normal maternal observations. An improvement in pain may have been falsely reassuring whilst representing ovarian necrosis. Prompt imaging may have been helpful in recognising AT. However, this woman was admitted in the middle of the night. Whilst there are international standards for the diagnosis and management of AT, out-of-hours ultrasound services are limited, and obstetricians and gynaecologists in training have varying degrees of expertise with gynaecological ultrasound [14].

Existing literature on AT in the third trimester of pregnancy presents common themes. Case reports have described AT across the breadth of this gestational period, from 28 weeks to 38 weeks [2,10,15-17]. The pain appears to be the predominant symptom [2,10,15-17] and is often described as “sudden”, “constant” or “sharp” and is more commonly right-sided [2]. The pain is almost always unilateral [10,15-17] and can be associated with nausea and/or vomiting. Symptom duration at presentation can vary from a few hours to one week. A raised WCC may be seen; however, fever is only present in a minority of cases [2].

In almost all cases described in the literature, ultrasound imaging was performed [2,10,15-17]. In this cohort, adnexal enlargement appears to be the most prevalent ultrasound feature [2]. In a subset of women with equivocal sonographic findings, MRI has also been used to guide management [10,15]. MRI can provide excellent soft tissue contrast, improving diagnosis of congestion, oedema, haemorrhage, and infarction, which are associated with AT [10]. The specific MRI finding associated with AT is fallopian tube thickening [18], which is found in 90% of cases [19].

Once AT is suspected in the third trimester, laparoscopy is often the surgical approach of choice [15-17]. Whilst laparotomy has also been described in the literature, expectant management is unusual [2]. Laparoscopy in the third trimester poses unique surgical and anaesthetic challenges, and the required expertise to perform this safely may not be widely available [20]. Laparo-endoscopic single-site surgery (LESS) avoids the negative effects associated with pneumoperitoneum in pregnancy, and this technique has been described for the management of AT in the third trimester [15]. As gestation approaches term, laparotomy with concurrent delivery is reasonable.

Cystectomy with de-torsion appears to be the most commonly reported surgical approach to AT in the third trimester [2,16,17]. However, salpingo-oopherectomy is also described often [2,10,15]. The wide use of salpingo-oopherectomy may in part be explained by diagnostic delay, leading to irreversible ovarian damage by the time surgery is performed. Where ovarian preservation is possible, oophoropexy may prevent recurrent AT in pregnancy [16]. Ovarian preservation appears to be even less common if a laparoscopic approach is used, with a paucity of cases describing ovary-sparing laparoscopic surgery for AT in the third trimester [17].

**Figure 3:**
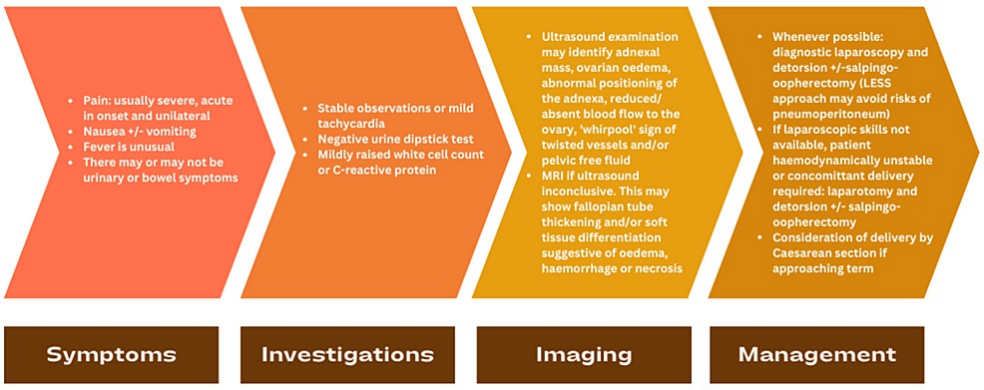
Schematic approach to adnexal torsion in the third trimester Image credits: Author (Subanhey Thiyagalingam)

There is no international consensus on the diagnosis and management of AT in the third trimester. On the basis of the existing literature, we propose a schematic approach to this rare but time-critical emergency (Figure 3).

## Conclusions

This case illustrates several learning points from a case of AT in the third trimester, which was missed. Whilst rare, this is a time-critical medical emergency, and clinicians must have a high index of suspicion for AT when assessing acute, severe pain in the third trimester, which cannot easily be explained by labour or other types of pathology. In particular, “loin to groin” pain is nonspecific and can be misleading. It should not automatically be ascribed to renal colic unless there is convincing evidence of ureteric calculi, which can include microscopic haematuria and/or radiological evidence.

Ultrasonography is an easily accessible and safe imaging modality that can be extremely helpful in the diagnosis of AT in pregnancy. There must be an increasing drive to ensure that obstetricians and gynaecologists in training can provide these skills out of hours. MRI should also be considered if ultrasound is inconclusive and the diagnosis remains unclear. Prompt surgical management of AT is essential, and surgery should not be delayed by imaging if the diagnosis is clear, as this can result in irreversible damage to ovarian tissue.
